# Three-Dimensional Quantification of Spheroid Degradation-Dependent Invasion and Invadopodia Formation

**DOI:** 10.1186/s12575-018-0085-6

**Published:** 2018-10-15

**Authors:** Cameron Goertzen, Denise Eymael, Marco Magalhaes

**Affiliations:** 1 Cancer Invasion and Metastasis Laboratory, Faculty of Dentistry, University of Toronto, Toronto, Canada; 2 Oral Pathology and Oral Medicine, Faculty of Dentistry, University of Toronto, 124 Edward Street, room 495, Toronto, ON M5G1G6 Canada; 3 Sunnybrook Health Sciences Centre, Toronto, ON Canada

**Keywords:** Spheroid invasion assay, Three-dimensional, Cancer invasion, Metastasis, Invadopodia, Immunohistochemistry, Basement membrane matrix, DQ-green BSA

## Abstract

Invadopodia are actin-rich, proteolytic structures that enable cancer cell to invade into the surrounding tissues. Several in vitro invasion assays have been used in the literature ranging from directional quantitative assays to complex three-dimensional (3D) analyses. One of the main limitations of these assays is the lack of quantifiable degradation-dependent invasion in a three-dimensional (3D) environment that mimics the tumor microenvironment. In this article, we describe a new invasion and degradation assay based on the currently available tumor spheroid model that allows long-term high-resolution imaging of the tumor, precise quantification, and visualization of matrix degradation and multichannel immunocytochemistry. By incorporating a degradation marker (DQ-Green BSA) into a basement-membrane matrix, we demonstrate the ability to quantitate cancer cell-induced matrix degradation in 3D. Also, we describe a technique to generate histological sections of the tumor spheroid allowing the detection of invadopodia formation in the 3D tumor spheroid. This new technique provides a clear advantage for studying cancer in vitro and will help address critical questions regarding the dynamics of cancer cell invasion.

## Background

Metastasis, the most devastating hallmark of cancer, is the spreading of cancer cells from the primary tumor to distant secondary sites of the body. Cancer cell migration and invasion of surrounding tissues are required steps during cancer metastasis [1–3]. The ability of cancer cells to migrate and invade through the basement membrane and extracellular matrix (ECM) is dependent on cellular structures known as invadopodia [4]. Invadopodia are cell membrane protrusions that bring together actin polymerization and matrix degradation to a focal area at the cell membrane [4]. Proteins identified as major contributors to actin polymerization and invadopodia formation include, but are not limited to, cortactin, Tks5, cofilin, N-WASP and Arp2/3 [5–8]. Image analysis and time dependent studies have revealed that these proteins co-localize in precursor invadopodia and can be used to identify early invadopodia formation [6]. Mature invadopodia result in degradation of the surrounding extracellular matrix through the delivery of proteases such as matrix-metalloproteinases (MMP)-2, − 9, or 14 [8, 9]. MMP-14, a membrane bound MMP, has demonstrated to play a crucial role in invadopodia-mediated invasion through the activation of other MMPs such as MMP-2 [9]. Co-localization of cortactin, Tks5 and MMP-14 indicates mature or active ECM degrading invadopodia and can be used for invadopodia identification [8–10]. In its native environment, cancer cells are presented with a complex three-dimensional (3D) environment of ECM, and most commonly develop into an organized 3D mass of cells. In this environment, not all cells are in contact with the stroma and some cells can degrade and reorganize the surrounding tissues, creating avenues for cancer cell migration and metastasis as we have shown before using 3D imaging of cancer cells [2, 6]. Hence, it is crucial to understand the underlying mechanisms of cancer cell invasion in a 3D environment so that preventative strategies targeting cancer metastasis may be developed.

To study cancer cell invasion, several in vitro assays have been developed and utilized. A common in vitro assay used to evaluate cancer invasion is the transwell invasion assay, two-dimensional assay used to quantitate invasion at a single cell level [11]. Briefly, the transwell invasion assay has two chambers separated by a porous membrane. A monolayer of cancer cells is seeded in the top chamber on the porous membrane covered in a basement membrane matrix. Although quantifiable, the process of invasion is not completely visualized and the cells invade in a single cell pattern, unidirectionally, from a top-down direction towards the chemoattractant [11–13]. To measure cancer cell invadopodia formation, the gelatin/fibronectin or collagen invadopodia assay is the most commonly used technique [7, 12]. Like the transwell invasion assay, a monolayer of cancer cells is placed on top of a layer of matrix and the ability of cancer cells to form invadopodia and degrade the basement membrane material is quantified. However, both the transwell invasion assay and gelatin invadopodia assay, like many 2D invasion assays, fail to replicate the conditions seen in the native 3D environment of the human body [14–20]. In the 3D environment of the human body, cancer cells start from a single source and invade in a multidirectional manner influenced by a plethora of signalling factors and modulators [1, 3]. Furthermore, the monolayer of cells used in the 2D assays does not reflect the dynamic interactions in-between cancer cells of the primary tumor and in-between tumor cells and the ECM. The primary tumor in a 3D environment demonstrates substantial variation in cellular morphology, gene expression patterns, cell differentiation, and cell-cell and cell-ECM adhesions that can be reproduced in 3D cultures [2, 14, 15, 17]. Although several in vivo models are available most are technically complex and cost prohibitive to be used on a regular basis [21].

The 3D spheroid invasion assay is an in vitro 3D culture technique that utilizes a tumor spheroid surrounded by extracellular matrix to mimic the native 3D environment of the human body [22]. The tumor spheroid is formed in a specialized non-adherent round bottom 96-well microplate with one spheroid formed per well. After spheroid formation, extracellular matrix is added to surround the tumor spheroid. Basement membrane matrix purified from murine Engelbreth-Holm-Swarm tumor or Collagen gels have been used in a variety of techniques for establishing both non-malignant and malignant cell growth that resembles the 3D in vivo environment [2, 8, 23, 24]. Several methods for generation of s tumor spheroids have been reported before and the strengths and weaknesses of these models has been reviewed before [24]. Spheroids are used to analyze tumor growth and behaviour, invasion and to test inhibitors. Particularly focusing on patterns of, spheroids provide a good model to study tumor invasion into collagen-based matrices in the 3D [22].*Vinci* et al. *2015* utilized imaging cytometer to rapidly and reproducibly measure the extent and rate of tumor spheroid invasion using the 3D spheroid invasion assay [22]. However, the 3D spheroid invasion assay has several limitations including the inability to distinguish between invasion and proliferation in contribution to spheroid volume [22]. It is suggested that concurrent proliferation assays with inhibitory or stimulatory agents be used to understand the effects on the cell line of interest [22, 25] and staining and imaging in high resolution has also been problematic due to specimen thickness and the overall fragile nature of the spheroid. Hence, the application of this technique has been limited when assessing the underlying mechanisms of cancer invasion. We have recently showed that degradation markers can be incorporated to the 3D matrix to monitor degradation using UMSCC1 cells [26]. Here, we use this new method to measure volume of matrix degradation and invadopodia formation in 3D spheroid samples, and use the same spheroids to generate formalin fixed, paraffin-embedded sections (FFPE) for FIHC and high-resolution imaging. We sucessfully applied this new method to study a breast cancer model (MDA-MB-231) and an oral cancer model (UMSCC1).

## Results

### Measuring 3D Degradation in Different Cancer Models

**Fig. 1:**
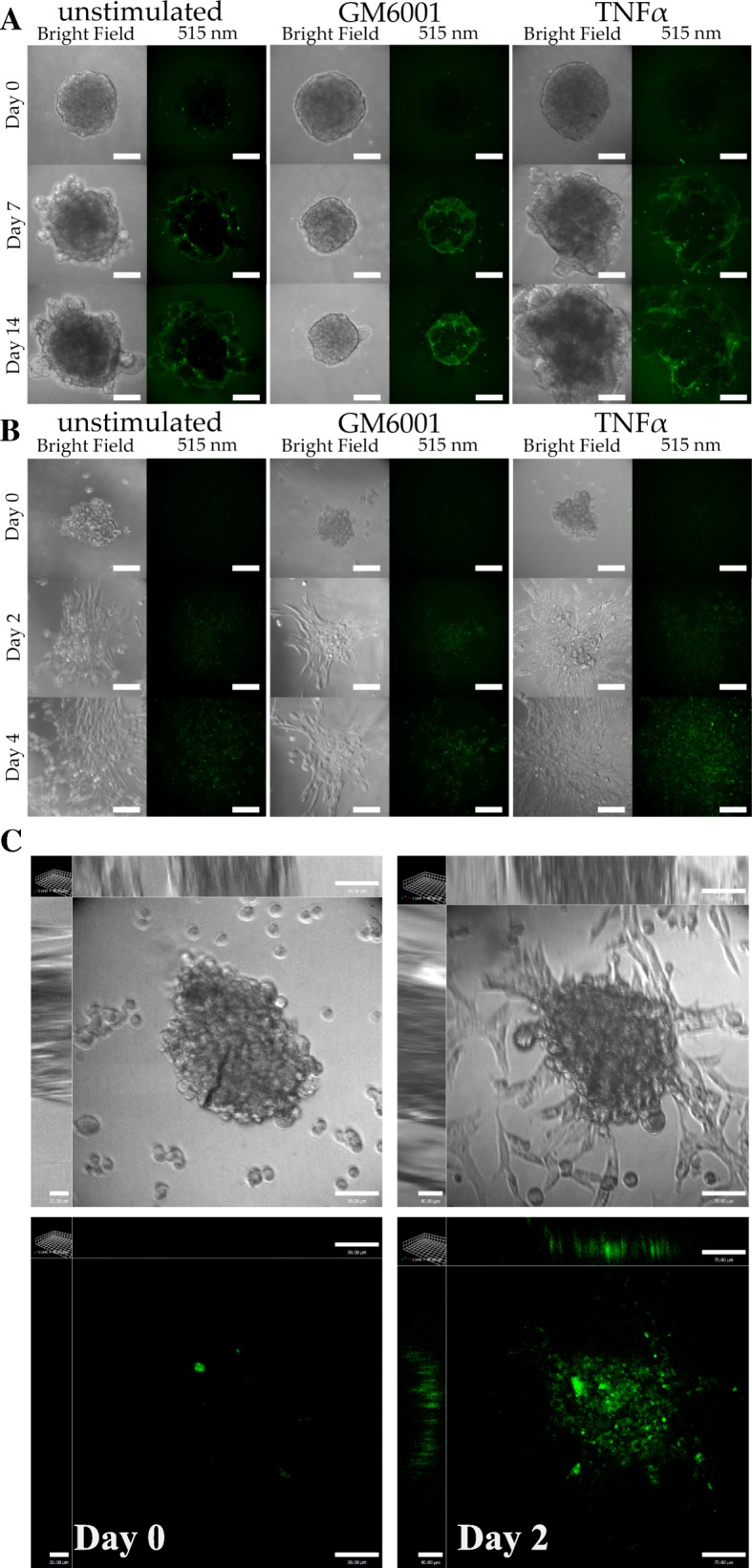
Representative images of spheroid Geltrex™ degradation. Representative confocal images of UMSCC1 (**a**) or MDA-MB-231 (**b**) spheroids causing Geltrex™ degradation signalling at Day 0, 7, and 14 or Day 0, 2, and 4, respectively. Spheroid volume was revealed through bright-field imaging and Geltrex™ degradation through 488 nm excitation/515 emission. **c** 3D images of MDA-MB-231 cells showing both a spheroid and a background of highly invasive cells that attached to the bottom of the slides. Scale bar, 100 μm. Images are representative of three repetitions

**Fig. 2:**
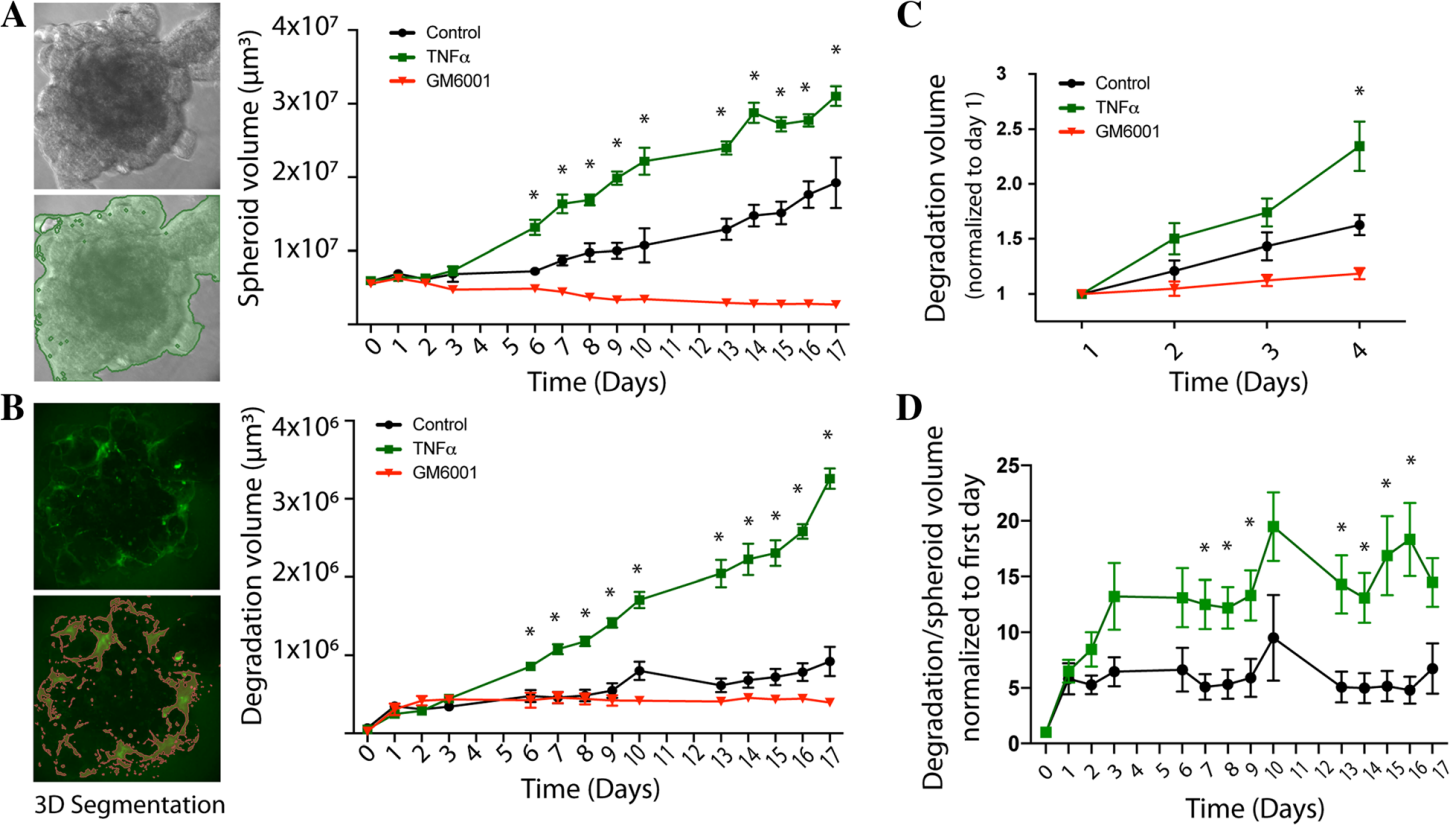
Spheroid Geltrex™ degradation analysis. **a** Representative software screenshot demonstrating spheroid volume annotation (left panels) and quantification of the tumor volume (graph). UMSCC1 Spheroid volume over a period of 17 days in the presence or absence of TNFα (10 ng/mL) or GM6001 (25 μM). **b** Representative software screenshot demonstrating Geltrex™ degradation volume in UMSCC1 spheroids over a period of 17 days in the presence or absence of TNFα (10 ng/mL) or GM6001 (25 μM). **c** MDA-MB-231 spheroid Geltrex™ degradation volume increase over a period of 4 days compared to Day 1 Geltrex™ degradation volume. Volumes are presented as mean per spheroid +/− SEM. Two-way ANOVA followed by Bonferroni post hoc test: * *P* < 0.01 for GM6001 vs. unstimulated; *P* < 0.01 TNFα vs. unstimulated; *n* = 3. **d** The degradation volume was divided by the spheroid volume and normalized to the first day of measurement. Two-way ANOVA followed by Tukey’s multiple comparison test: *P < 0.01

In virtue of the advantages provided by the 3D spheroid invasion assay, we set out to develop a method to address some of the limitations of the assay and use it to assess the underlying mechanisms of cancer invasion. To measure basement membrane matrix degradation, we incorporated the fluorogenic substrate, DQ-Green BSA, into the Geltrex™ surrounding the tumor spheroid. DQ-Green BSA is a derivative of bovine serum albumin labeled with BODIPY dye, a self-quenched fluorogenic substance. Upon degradation by proteases released by the tumor spheroid, DQ-Green BSA fragments are fluorescent and can be imaged using fluorescent microscopy [27]. This allows the quantification and visualization of 3D Geltrex™ degradation. As seen in Fig. 1 we were able to generate tumor spheroids of breast cancer cell line MDA-MB-231 (Fig. 1b) and oral cancer cell line UMSCC1(Fig. 1c) in Geltrex/DQ-green BSA matrix. We next wanted to use this model to determine if we can measure differences in degradation after stimulation of invasion in both cells. We recently used this model to show that TNFα increases degradation-dependent invasion in UMSCC1 cells [26], therefore we replicated the experiments here using using TNFα to promote invasion and compare tumour spheroid invasion in both cancer models. TNFα treatment increased the rate of tumor growth (Fig. 2a) and matrix degradation of both cell lines (Fig. 2b-c). To control for degradation-dependent spheroid invasion, we added the GM6001 protease inhibitor to the matrix and to the media which resulted in inhibition matrix degradation and spheroid growth in both cells tested (Fig. 2). Importantly, TNFα treatment induced a significantly larger increase in spheroid degradation compared to tumor growth suggesting that TNFα may primarily affect degradation mechanisms and not cell growth rate (Fig. 2d). This could only be evaluated because of the addition of the matrix degradation marker. Using this model, we were able to culture tumor spheroids from UMSCC1 cells for up to 14 days and the highly invasive MDA-MB-231 cells for 4 days. UMSCC1 showed a predominantly collective pattern of invasion with occasional single cell extension while MDA-MB231 cells showed a predominately single cell pattern of invasion quickly reaching the bottom surface of the culture dish (Fig. 1c). During the quantification of the degradation signal, a persistent DQ-Green BSA fluorescent signal was seen at the periphery of the spheroid in GM6001-treated samples. We believe that since the spheroid cannot degrade the surrounding matrix, any leftover degradation activity that is not inhibited by GM6001 is confined to the periphery of the spheroid and accumulates creating the impression of increased degradation.

### Measuring 3D Invadopodia-Dependent Invasion in High Resolution

**Fig. 3:**
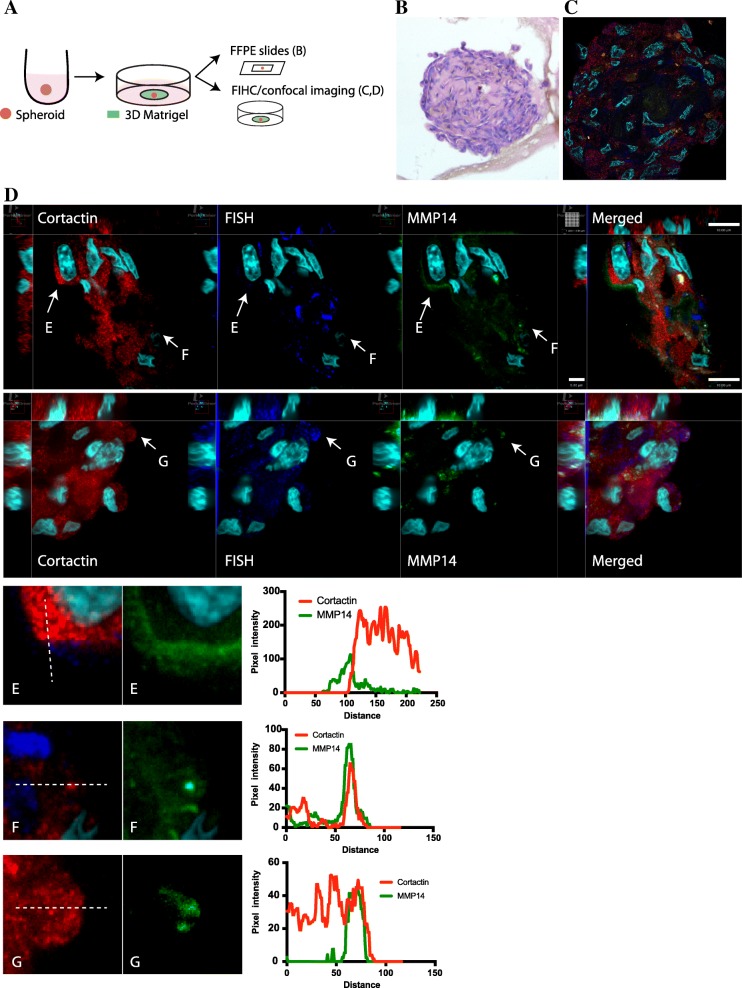
Spheroid immunohistochemistry and invadopodia formation. **a** Summary of the protocol for generation of spheroid and data analysis. **b** Representative images of sectioned MDA-MB-231 spheroid stained with Hematoxylin and eosin imaged with light-microscopy (40× magnification). The adjacent spheroid section was incubated for antibodies targeting cortactin (red), Tks5 (dark blue), and MMP-14 (green) and counter-stained with DAPI (light blue) and images using super-resolution **(c)**. **d** MDA-MB-231 tumor spheroids were stained with cortactin (Red), Tks5 (blue) and MMP-14 (green) and imaged using confocal microscopy. Top images represent a collective area of invasion and the bottom panel shows single cells extending away from the spheroid (arrows). Images are representative of 3 repetitions. Scale bar, 10 μm. Panels **(e-g)** are representative areas of MMP14 accumulation at the extracellular space, invadopodia and cell periphery respectively. Graphs show the pixel intensity distribution over the dashed lines shown on the left panels

To assess tumor spheroid invadopodia formation, we created 2 approaches: First, we adapted the spheroid technique to generate 5-μm sections of the spheroid for generation of FFPE sections to be stained using conventional hematoxylin-eosin methods (Fig. 3a). Second, we stained both FFPE sections and native spheroids in 3D matrices and analyzed them using confocal and super-resolution microscopy. As seen in Fig. 3b, we were able to localize, embed and section the spheroids for further histological analysis. Spheroids were stained for invadopodia markers (cortactin, Tks5, MMP-14) and imaged using conventional confocal microscopy (3D) to detect areas of invadopodia formation. There was a significant increase in MMP14 expression at the matrix adjacent of the invasive cells (Fig. 3d/e) as well as focal aggregates at invadopodia (Fig. 3d/f) and the cell periphery (Fig. 3d/g), supporting the results showing increased matrix degradation at the periphery of the spheroid near areas of invasion (Figs. 1 and 2). Finally, tumor spheroid sections were stained using Hematoxylin and Eosin (H & E) to analyze and compare tumor morphology which is currently a major limitation of 2D invasion methods (Fig. 3b). Thus, the 3D tumor spheroid degradation/invasion assay protocol outlined here can serve as a basis for the analysis of 3D cancer degradation-dependent invasion and determination of mechanisms underlying cancer cell invasion such as invadopodia formation.

## Discussion

The main goal of an in vitro cancer invasion assay is to replicate as much as possible the native environment of the tumor while providing quantifiable, reproducible results. Here we adapted a novel 3D spheroid degradation assay [26] to analyze matrix degradation, invasion, tumor growth and invadopodia formation all in a 3D tumor spheroid environment. Most importantly, the addition of a degradation marker allowed the quantification of 3D degradation and a better understanding of tumor growth.

During the development of the assay we determined that the number of cells placed in each well of the 96-well plate, will vary according to cell line used and the desired size of spheroid to be formed. For the use of cell line UMSCC1, 5,000 cells produced a spheroid of 200–300 μm diameter before placement of Geltrex™. Creating a spheroid of larger size, through increased number of cells place in each well, resulted in a spheroid that grew in volume that exceeded the field of view of the 10× magnification microscope used to image. Visualization of the spheroid with the unaided eye is possible with adequate lighting, though not necessary for aspiration with a well place pipette tip.

Regarding the cell lines used in our experiments, the UMSCC1 cell line produced consistent size spheroids over each repetition allowing for direct comparisons between spheroid volume and Geltrex™ degradation. However, the MDA-MB-231 cell line, possibly due to decreased cell-to-cell adhesion, produced spheroids of varying size from occasional splitting of spheroids to 2–3 smaller cell clusters during Geltrex™ placement. Importantly, the incubation time required before spheroid formation varies between tumor cell lines and is suggested to correlate with cell-to-cell adhesion capacity. The UMSCC1 cell line of epithelial origin, demonstrates a high capacity for cell-to-cell adhesion and spheroids were visibly formed at 24-h cell incubation. The MDA-MB-231 cell line demonstrates a low capacity for cell-to-cell adhesion and at 24 h’ incubation, visible spacing between multiple cell clusters was apparent. At 72 h, the MDA-MB-231 cell line was able to form a single spheroid without visible inter-cellular spacing. Length of experimentation dependent on invasive capacity of cell line. The more invasive MDA-MB-231 cell line invaded beyond 10× field of view at day 5 while less invasive UMSCC1 cell line invaded beyond 10× field of view after 15 days of observation. The differences in culture time and pattern of invasion explains the observed lower signal of BSA-Green in the MDA-MB-231 cells compared to UMSCC1 cells.

The number of wells used to form spheroids will depend on the desired number of replications per condition. For example, in Fig. 1, three conditions were observed per cell line. For each condition, we created/examined 3 spheroids. Hence, for one cell line we created 9 spheroids (3 conditions × 3 spheroid repeats). However, it is recommended that “back-up” spheroids are formed in the event that a spheroid is mal-formed (not spherical, visible cell debris). From experience, approximately 1 in 10 spheroids were mal-formed before the addition of Geltrex™.

Using a second 96-well plate enables the formation of a bottom layer of Geltrex™. The bottom layer of Geltrex™ ensures that the spheroid will always be surrounded by Geltrex™ during the desired period of experimentation. In the Geltrex™ degradation analysis set-up, the added bottom layer of Geltrex™ is omitted so that the spheroid will be at the lowest level of the well, required so the spheroid will be within the limited focal range of the confocal microscope used for live daily images. Unfortunately, omitting the bottom layer, limits the period of Geltrex™ degradation analysis that can be performed. Using the highly invasive MDA-MB-231 cell lines, by 5 days of invasion, cells have invaded through the bottom layer of Geltrex™ and formed a monolayer of cells on the well bottom.

Transferring the spheroids from the Corning spheroid round bottom 96-well microplate to the 96-well flat bottom plate is performed to ensure that during imaging, the spheroid will always be within the limited focal range of the microscope. If Geltrex™ is added directly to the round-bottom well, often the spheroid is displaced from the lowest point of the round bottom, and moving above the focal range of the microscope required for imaging. By using a flat-bottom well plate, the spheroid will always be within the microscope focal range.

Our method also allows the quantification of degradation-dependent and independent invasion. Our data suggests that spheroid volume and degradation and two complementary datasets that need to be evaluated together, particularly because: 1- The increase in degradation (Fig. 2b) is higher than the increase in tumor volume (Fig. 2a). 2- Tumor volume did not increase over time with GM6001 inhibition in (Figs. 1 and 2), although GM6001 treatment does not directly affect cell growth. Therefore, we believe that before a tumour can increase in volume, matrix remodelling is needed and this may or may not require significant degradation of the matrix, depending on the tumor model used. Our model allows the distinction between these two scenarios.

## Conclusions

We have developed a new reproducible method to measure degradation in 3D using a tumor spheroid model. With this new method, we were able to quantify matrix degradation, invasion and analyze invadopodia formation in 3D tumor spheroids in high-resolution. This is a significant advance for cancer research since it overcomes some of the current challenges we encounter while using in vitro models to study invasion. Our method will allow a more comprehensive evaluation of cancer invasion by allowing direct quantification of spheroid degradation-dependent invasion while allowing for high-resolution image at the cell level.

## Methods

### Materials

#### Formation and Culture of Tumor Spheroids

T75 cell culture flask (ThermoFisher). Cancer cell line (human breast adenocarcinoma: MDA-MB-231 or oral squamous cell carcinoma (OSCC): UMSCC1). Cell culture growth media (Dulbecco’s Modified Eagle Medium (DMEM; Thermo Fisher Scientific) supplemented with 10% (*v*/v) fetal bovine serum (FBS; Thermo Fisher Scientific), non-essential amino acids (100 nmol/L; Thermo Fisher Scientific), penicillin and streptomycin liquid (100 mg/mL; Thermo Fisher Scientific). Pipette tips (2–200 μl, 100–1000 μl). Phosphate Buffered Saline (PBS) (Sigma-Aldrich D8537). Trypsin-EDTA (0.25% Trypsin with EDTA 4Na) (Life Technologies). Falcon 15 ml conical centrifuge tubes (Fisher Scientific). Hemocytometer. Corning spheroid microplates (96 well, black/clear bottom round, Ultra-Low Attachment surface).

#### Tumor Spheroid Invasion Assay Set-up

96-Well x 400uL Cell Culture Microplate (167314, Capital Scientific). Geltrex™ LDEV-Free Reduced Growth Factor Basement Membrane Matrix (A1413202, Life Technologies). Geltrex™ basement membrane matrix is liquid at low temperatures (4 °C) but solidifies quickly at room temperature. Contact with items that are room temperature may cause Geltrex™ to prematurely solidify, preventing proper placement of spheroid in Geltrex™.1.5 ml microcentrifuge tubes. DQ-Green BSA (D12050, Thermo Fisher). Pipette tips (0.1–2.0 μl, 2–200 μl, 100–1000 μl). Ligands and inhibitors TNFα (#8902sf, Cell signaling) and GM6001 (M5939 Millipore)). Centrifuge with 96-well plate attachments. Cell culture growth media (Dulbecco’s Modified Eagle Medium (DMEM; Thermo Fisher Scientific) supplemented with 10% (*v*/v) fetal bovine serum (FBS; Thermo Fisher Scientific), non-essential amino acids (100 nmol/L; Thermo Fisher Scientific), penicillin and streptomycin liquid (100 mg/mL; Thermo Fisher Scientific).

#### Spheroid Geltrex™ Degradation Analysis

Leibowitz’s L-15 Medium, no phenol red (21083027, ThermoFisher) supplemented with 10% (v/v) fetal bovine serum. L-15 medium is buffered by phosphates and free base amino acids instead of sodium bicarbonate, alleviating the need for 5% CO_2_ during the live cell imaging. The absence of phenol red in L-15 is used to prevent phenol red autofluorescence during immunofluorescence image acquisition. Plastic paraffin film (Parafilm). Cell culture growth media (Dulbecco’s Modified Eagle Medium (DMEM; Thermo Fisher Scientific) supplemented with 10% (v/v) fetal bovine serum (FBS; Thermo Fisher Scientific), non-essential amino acids (100 nmol/L; Thermo Fisher Scientific), penicillin and streptomycin liquid (100 mg/mL; Thermo Fisher Scientific). Ligands and inhibitors (#8902sf, Cell signaling) and GM6001 (M5939 Millipore)). Spinning disk confocal microscopy (Quorum spinning disk confocal, Leica DMIRE2). We used Volocity 6.3 to analyze the 3D images generated from the spheroids. Using Volocity we were able to accurately select the BODIPY-green signal using the standard deviation of the pixel intensity and analyze volume, intensity, shape (circularity, roundness).

#### Immunohistochemistry Spheroid Processing, Staining and Invadopodia Analysis

4% Paraformaldehyde (PFA). Phosphate Buffered Saline (PBS). Ethanol (50%, 70%, 80%, 95%, 100%). Ethanol: Xylene (2:1, 1:1; 1:2). Xylene (100%). Xylene: Paraffin (2:1, 1:1, 1:2). Paraffin (100%). Paraffin oven. Paraffin cassettes. Rotary Microtome (Leica RM2125 RTS). Superfrost Plus Microscope Slides 15x75x1.0 mm (Fisherbrand, # 12–550 15). TRIS-buffered saline (TBS) plus 0.025% Triton X-100. 1% (Bovine serum albumin) BSA in TBS. Primary Antibodies (Cortactin (ab84208, Abcam), Tks5 (ab118575, Abcam Biotechnology), MMP-14 (ab56307, Abcam). Secondary Antibodies (Donkey F(ab’)2 Anti-Goat IgG H&L (Alexa Fluor 647) (ab150139, Abcam), Donkey F(ab’)2 Anti-Rabbit IgG H&L (Alexa fluoro-568) (ab175694, Abcam), Donkey F(ab’)2 Anti-Mouse IgG H&L 488 (ab150155, Abcam). DAPI (NucBlue Fixed Cell Ready Probes reagent, Life technologies - Catalog# R37606). Distilled water. Harris Hematoxylin solution. Lithium carbonate solution. 5% Eosin solution. Mounting medium (ProLong diamond Antifade Mount, Life technologies, #P36961). Coverslips (22mmX50mm 1.5 oz. cover class, Electron Microscopy Sciences Sciences. Catalog#72204–04. ZEISS ELYRA PS.1 super-resolution microscopy, Quorum Spinning Disk Confocal microscope. Imaging analyzing software: ZEISS ZEN 2.3 and Volocity 6.3.

### Methods: Generation of Tumor Spheroids

1. In a T75 culture flask, culture tumor cell monolayers in cell medium (Dulbecco’s Modified Eagle Medium (DMEM) supplemented with 10% (*v*/v) fetal bovine serum (FBS)) in culture incubator (at 37 °C with 5% CO_2_) until cell confluency of 60–80%. In Fig. 1, human breast adenocarcinoma cell line, MDA-MB-231, and oral squamous cell carcinoma (OSCC) cell line, UMSCC1, were used.
2. Using phosphate buffered saline (PBS), wash tumor cell monolayers to remove culture medium (10 ml per 75 cm^2^).
3. Trypsinize cells (2 ml per 75 cm^2^) until cells have detached (4–10 min). Once cells have begun lifting off flask, add DMEM medium contacting 10% (v/v) FBS (8 ml) to inactivate trypsin. Transfer cell suspension into 15 ml conical tube.
4. Centrifuge cell suspension at 300 RCF for 5 min.
5. Aspirate cell medium supernatant. Using a P1000 pipette, re-suspend cell pellet in 1 ml of DMEM medium contacting 10% (v/v) FBS.
6. With a hemocytometer, determine cell concentration of cell suspension. Dilute cell suspension with DMEM medium contacting 10% (v/v) FBS to obtain a concentration of 50,0000 cells/ ml.
7. Transfer 5,000 cells (100 μl) from cell suspension to each well of a Corning spheroid round bottom, Ultra-Low Attachment 96-well microplate.
8. Transfer 96-well microplate to cell culture incubator (at 37 °C with 5% CO_2_) and incubate cells until spheroids form (approximately 24–72 h).

### Tumor Spheroid Invasion Assay Set-up

1. Preparation: cool pipette tips (0.1–2.0 μl, 2–200 μl, 100–1000 μl) and 96-well flat bottom plate at 4 °C overnight.
2. Thaw Geltrex™ basement membrane matrix on ice until Geltrex™ is liquid (45–60 min).
3. Prepare 1.5 ml microcentrifuge tubes that will be used for each spheroid condition by labeling and placing on ice until cool.
4. When Geltrex™ is liquid, distribute Geltrex™ into 1.5 ml microcentrifuge tubes for each condition of interest so that each spheroid well will contain 100 μl of Geltrex™. In other words, if the condition of interest will have 3 spheroids, distribute 300 μl of Geltrex™ into designated microcentrifuge tube for that condition using chilled pipette tips.

- For spheroid Geltrex™ degradation analysis, add Green-BSA to Geltrex™ to achieve concentration of 30 μg/ml for each condition.

5. If using ligands or inhibitors, add 1.2× required concentration to Geltrex™ for each condition of interest. For Fig. 1, ligand TNFα (10 ng/ml) and inhibitor GM6001 (25 μM) were used.
6. For spheroid Geltrex™ degradation analysis proceed to step 3.2.7. For IHC invadopodia analysis, add 50 μl of prepared Geltrex™ to each well of chilled 96-well flat bottom plate using chilled pipette tips. Perform steps 3.2.9 and 3.2.10 before proceeding to step 3.2.7.
7. Remove Corning spheroid round bottom, Ultra-Low Attachment 96-well microplate containing spheroids from incubator. Transfer spheroids from the round-bottom 96-well plate to the flat-bottom 96-well plate. To transfer spheroids, using a P200, place tip of pipette at the lowest depth of the round-bottom well and aspirate 20 μl of media containing spheroid. Transfer spheroid to well of 96-well flat bottom plate. Repeat, until all spheroids have been transferred. Confirm spheroid transfer under microscope.
8. For spheroid Geltrex™ degradation analysis, add 100 μl of prepared Geltrex™ to each well. For IHC invadopodia analysis, add 50 μl of prepared Geltrex™ to each well to form top layer. Ligands and inhibitors are added at concentrations 1.2× to account for dilution from media when adding spheroid
9. Centrifuge 96-well plate at 300 RCF for 5 min at 4 °C to remove any bubbles formed in the Geltrex™ during pipetting.
10. Incubate 96-well plate containing Geltrex™ at 37 °C for 30 min to solidify Geltrex™.
11. Add 100 μl of cell culture medium containing desired concentrations of ligands or inhibitors, if used, to each well. Incubate spheroids at 37 °C with 5% CO_2_ for desired length of experimentation, removing and adding new media every 3–5 days.

### Spheroid Geltrex™ Degradation Analysis

1. At desired time (s) from initial placement of spheroids in Geltrex™, gently remove 100 μl of cell culture medium and replace with 100 μl of Leibowitz’s L-15 phenol red free medium supplemented with 10% (*v*/v) fetal bovine serum.
2. Wrap periphery of 96-well plate with plastic paraffin film to ensure sterility.
3. Using confocal microscopy, locate and image the spheroid of each well. Images are taken with bright field channel and 505 nm excitation to visualize Green-BSA 515 nm emission signaling produced from spheroid Geltrex™ degradation. Utilizing z-stack image acquisition (1 μm slices), create a stack of images from the bottom of the spheroid to the top using Green-BSA signaling as measure.
4. Remove paraffin film and replace L-15 phenol red free medium with 100 μl of cell culture medium containing desired ligands or inhibitors. Incubate spheroids at 37 °C with 5% CO_2_.
5. Repeat previous steps (3.3.1–3.3.4) at each desired time points of experimentation to visualize spheroid growth and degradation (Fig. 1).
6. To analyze spheroid growth and degradation volume, use image analysis software (Volocity 6.3). To measure total spheroid volume, use bright-field images and set software parameters to trace total area of spheroid for each image of the z-stack (Fig. 2a). Similarly, to measure total Geltrex™ degradation volume, use 515 nm images and set software parameters to measure areas of green fluoresce signal for each image of the z-stack. The spheroid volume and Geltrex™ degradation volume is calculated by the software as a single μm^3^ value per z-stack and can be reported for each day for comparison (Fig. 2b and c). Alternatively, if initial sizes of spheroids vary, volume of Geltrex™ degradation can be normalized to the first day of volume acquisition (Fig. 2d).

### Immunohistochemistry, Spheroid Processing, Staining and Invadopodia Analysis

1. At desired time from initial placement of spheroids in step 3.2.11, gently remove 100 μl of cell culture medium using P200.
2. Wash each well with PBS (100 μl).
3. Add 150 μl of room temperature 3.7% paraformaldehyde to each well and place 96-well plate at 4 °C overnight.
4. Remove paraformaldehyde using P200 and wash each well with PBS (100 μl) three-times. Add 100 μl of PBS and store at 4 °C until proceeding with step 3.4.5.
5. Remove PBS and dehydrate tissue using 100 μl per well in sequence of the following solutions: 50% ethanol (10 min), 70% ethanol (10 min), 80% ethanol (10 min), 95% ethanol (10 min), 100% ethanol (10 min), 100% ethanol (10 min), and 100% ethanol (10 min).
6. Transfer dehydrated Geltrex™ containing spheroid to 1.5 ml microcentrifuge tube.
7. Exchange ethanol with xylene using 100 μl per well in sequence of the following solutions: 2:1 ethanol: xylene (15 min), 1:1 ethanol: xylene (15 min), 1:2 ethanol: xylene (15 min), 100% xylene (15 min), 100% xylene (15 min), and 100% xylene (15 min).
8. Exchange xylene with paraffin, in paraffin oven set for 54–58 °C, using 100 μl per well in sequence of the following solutions: 2:1 xylene: paraffin (30 min), 1:1 xylene: paraffin (30 min), 1:2 xylene: paraffin (30 min), 100% paraffin (2 h), and 100% paraffin (overnight).
9. Embed in new paraffin and orient tissue in paraffin cassette for microtome sectioning.
10. Chill paraffin-embedded tissue blocks on ice before sectioning.
11. Fill a water bath with ultrapure water (45 °C).
12. Following the microtome manufacturer’s instructions, insert paraffin block and section at thickness of 5 μm.
13. Place sections in water bath to float and lift individual sections out of water bath using glass microscope slide.
14. Dry slides overnight at 37 °C.
15. With selected slides, wash with TRIS-buffered saline (TBS) plus 0.025% Triton X-100 for 5 min in gentle slide shaker, repeat three-times.
16. Block with 1% (Bovine serum albumin) BSA in TBS for 2 h at room temperature.
17. Apply primary antibody diluted in TBS plus 1% BSA overnight at 4 °C. For invadopodia formation analysis, use primary antibodies for cortactin, Tks5, and MMP-14.
18. Wash slides with TBS plus 0.025% Triton X-100 for 5 min in gentle slide shaker, repeat three-times.
19. Apply secondary antibody diluted in TBS plus 1% BSA overnight at 4 °C.
20. Wash slides with TBS plus 0.025% Triton X-100 for 5 min in gentle slide shaker, repeat three-times.
21. Counterstain with DAPI (1 μg/ml) in TBS plus 1% BSA for 60 min at room temperature. Cover from ambient light.
22. Wash slides with TBS plus 0.025% Triton X-100 for 5 min in gentle slide shaker, repeat three-times.
23. Mount using mounting medium and add coverslip. Dry at room temperature for 24 h before imaging.
24. Using confocal microscopy, locate and image the spheroid of each slide section. Images are taken with 488 nm, 568 nm, 647 nm, and 405 nm excitation to visualize MMP-14, Cortactin, Tks5, and DAPI, respectively. Utilizing z-stack image acquisition (0.1 μm slices), create a stack of images from the bottom to top of the spheroid section for invadopodia analysis.
25. To analyze invadopodia formation and maturation, use an image analysis software capable of handling 3D stacks (e.g. ZEISS ZEN 2.3, Imaris or Volocity 6.3). Co-localization of invadopodia markers cortactin and Tks5 (Fig. 3) are an indication for precursor invadopodia formation. Co-localization of cortactin and MMP-14 are representative of mature, invading invadopodia (Fig. 3).

### Hematoxylin and eosin staining

1. For spheroid visualization, hematoxylin and eosin (H & E) staining can be performed concurrently following step 3.4.14.
2. Using unstained spheroid section adjacent to section used for step 3.4.15, deparaffinize section using 100% xylene (3 min), repeat three times.
3. Re-hydrate in sequence 100% ethanol (2 min, three times), 95% ethanol (2 min, twice), and 70% ethanol (2 min, twice).
4. Wash in distilled water (2 min, twice).
5. Place slide in Harris Hematoxylin solution (7 min).
6. Wash in distilled water (2 min).
7. Differentiate in 0.5–1% acid alcohol (3 dips).
8. Wash in distilled water (1 min).
9. Counterstain in Lithium carbonate solution (1 min).
10. Wash in distilled water (2 min, twice)
11. Place in 5% Eosin solution (1 dip)
12. Dehydrate in sequence 95% ethanol (30 s, twice) and 100% ethanol (30 s, twice).
13. Place in 100% xylene (2 min, three times).
14. Mount using 2–3 drops of mounting medium and add coverslip. Dry at room temperature for 24 h before imaging.
15. Image H&E stained spheroid section using light microscopy (Fig. 3).
