# Correction to: Three-Dimensional Quantification of Spheroid Degradation-Dependent Invasion and Invadopodia Formation

**DOI:** 10.1186/s12575-019-0100-6

**Published:** 2019-05-21

**Authors:** Cameron Goertzen, Denise Eymael, Marco Magalhaes

**Affiliations:** 1 Cancer Invasion and Metastasis Laboratory, Faculty of Dentistry, University of Toronto, Toronto, Canada; 2 Oral Pathology and Oral Medicine, Faculty of Dentistry, University of Toronto, 124 Edward Street, room 495, Toronto, ON M5G1G6 Canada; 3 Sunnybrook Health Sciences Centre, Toronto, ON Canada

**Correction to: Biological Procedures Online (2018) 20:20**

**https://doi.org/10.1186/s12575-018-0085-6**

It has come to the authors’ attention that the representative image of the unstimulated UMSCC1 spheroid at Day 1 in Fig. 1a was selected from the wrong data set. The image in the original article [1] was taken from a data set for another study by the authors [2]. The revised Fig. 1 including a representative image of the unstimulated UMSCC1 spheroid at Day 1 taken from the correct study data set is available in this erratum.

**Fig. 1:**
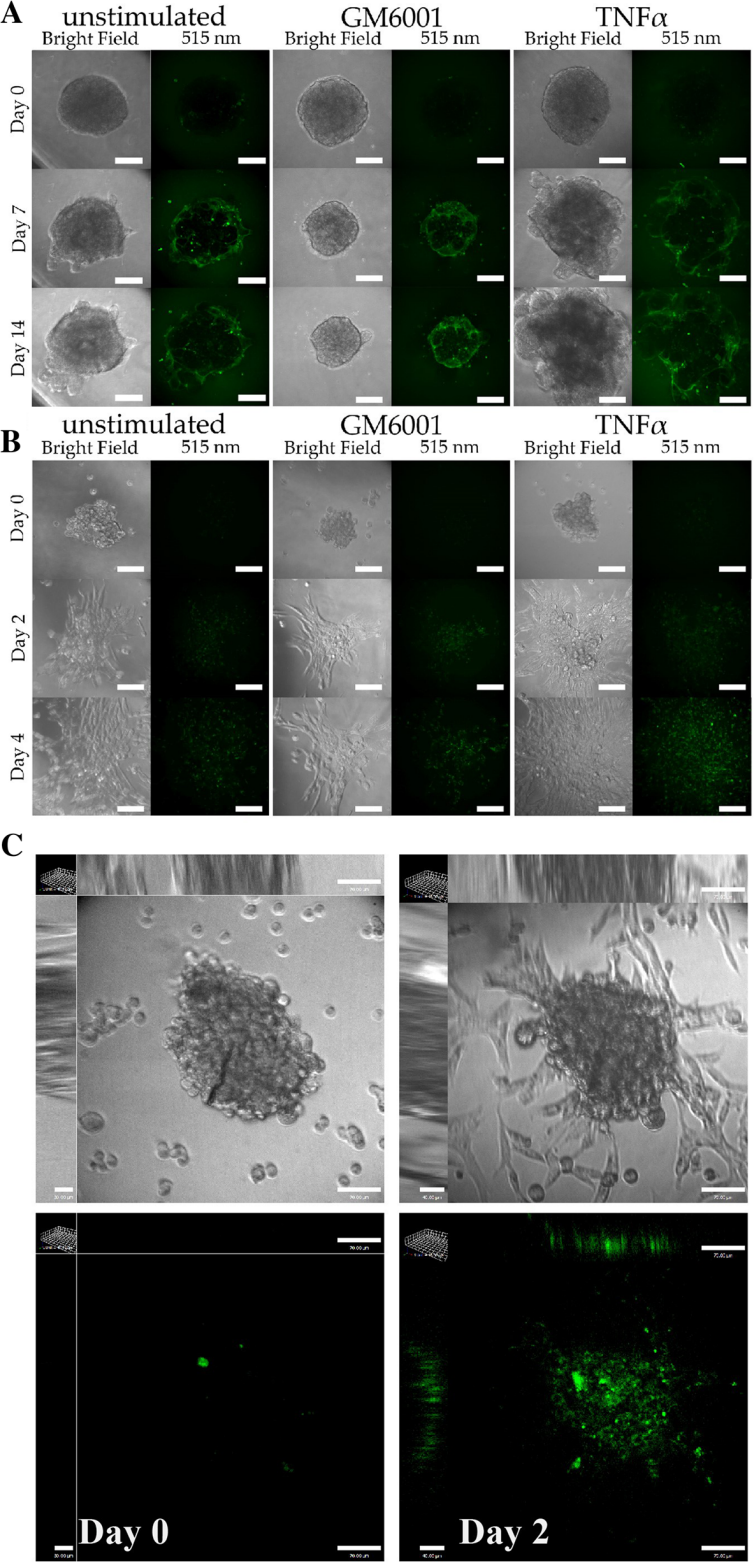
Representative images of spheroid Geltrex™ degradation. Representative confocal images of UMSCC1 (**a**) or MDA-MB-231 (**b**) spheroids causing Geltrex™ degradation signalling at Day 0, 7, and 14 or Day 0, 2, and 4, respectively. Spheroid volume was revealed through bright-field imaging and Geltrex™ degradation through 488 nm excitation/515 emission. **c** 3D images of MDA-MB-231 cells showing both a spheroid and a background of highly invasive cells that attached to the bottom of the slides. Scale bar, 100 μm. Images are representative of three repetitions
